# Feasibility of radiology online structured oral examination for undergraduate medical students

**DOI:** 10.1186/s13244-022-01258-9

**Published:** 2022-07-18

**Authors:** Fawaz Alharbi, Ali Alamer

**Affiliations:** Department of Radiology, College of Medicine, Qassim University, Buraidah, 6655-51452 Saudi Arabia

**Keywords:** Radiology, Online, Structured oral examination, Summative assessment, Medical students

## Abstract

**Background:**

Online summative assessment has emerged during the COVID-19 pandemic as an alternative to traditional examinations, bringing opportunities and challenges. The study aims to evaluate the feasibility and effectiveness of online structured oral examination (SOE) in radiology clerkships. The study identifies measures taken to successfully implement online SOE and minimize chances of cheating. It also discusses the challenges encountered and how they were addressed.

**Methods:**

SOE percent scores of fourth-year medical students from two institutions were correlated with students’ grade point average (GPA). The scores were compared among different institutions, students’ genders, students’ batches, examination versions, and examiners with different experience levels. Students’ perceived satisfaction and concerns were captured using anonymous self-administered questionnaire. Technical problems and success rate of SOE implementation were recorded. Results were analyzed using descriptive and inferential statistics.

**Results:**

A total of 79 students participated in the study, out of which 81.0% (*n* = 64) responded to the survey. SOE scores showed poor positive correlation with the students’ GPAs (*r* = 0.22, and *p* = .09). Scores showed no significant difference between the two institutions or genders. Scores were also not significantly different between students who were examined by junior or senior examiners. All but one version of examination showed no significant difference in students’ scores. No significant difference was observed in students’ scores between each two subsequent batches who were exposed to the same examination version.

**Conclusion:**

Online summative SOE is a feasible alternative whenever face-to-face SOE could not be implemented provided that appropriate measures are taken to ensure its successful execution.

## Key points

- Online SOE is feasible alternative whenever face-to-face SOE could not be implemented.
- Backup strategies ensure smooth execution of online SOE and reduce students’ anxiety.
- Multiple exam versions with questions testing higher cognitive abilities can limit cheating.
- A detailed scoring guide improves examination fairness and makes one examiner sufficient.

## Background

Oral examination has been long used in undergraduate and postgraduate medical assessment. It is superior to written examination in assessing higher cognitive domains as it tests “knows how” on Miller’s hierarchical model for the assessment of clinical competence, a level higher than “knows” which is usually tested in written examinations [1]. Nevertheless, there are several problems associated with traditional unstructured oral examination including high subjectivity, low overall and inter-rater reliabilities, low validity, case specificity problem, and examiner’s and examinee’s biases [2–4]. To solve some of these issues, structured oral examination (SOE) was introduced. In SOE, the questions and the correct answers with their scores are predetermined to ensure standardized examination process and consistency from one examiner to another and from one examinee to another. This resulted in improved overall and inter-rate reliability and showed acceptable validity [2, 3].

The rapid pace of technological advancements has impacted our lives and work environment. Of particular interest to medical educators, undergraduate medical education has shown increasing utilization of electronic (e)-learning and e-assessment resources [5]. This has brought opportunities for innovation in assessment along with several risks and challenges [6]. Driven by such communication and technological advancements, the nature of radiology which lends itself to technology, and the COVID-19 pandemic restrictions, we found it an opportunity to explore various online assessment tools. Among these was the online SOE, which is best suited for assessing interpretation skills. Justaniah et al. have recently reported their experience with online oral examination for interventional radiology fellowship programme [7], while others have reported on online mock oral examinations in radiation oncology and in vascular surgery [8, 9].

This article discusses the solutions implemented by the radiology department to achieve a successful and smooth implementation of online SOE, and strategies used to minimize chances of cheating. It also elaborates on the challenges encountered along the way, and how they were dealt with. Our experience may help others plan and tailor their online SOE and prepare for possible upcoming challenges.

## Methods

### Radiology clerkship

Radiology clerkship is a two-credit hour per week mandatory requirement in the fourth year of a five-year Bachelor of Medicine and Surgery programme in our faculty. The compulsory clerkship is also conducted by our faculty at another regional medical college as part of the collaboration between the two academic institutions. The learning objectives and teaching methods of the radiology clerkship were discussed in detail in our prior article [10].

Prior to the COVID-19 pandemic, students’ evaluation was based on two on-campus face-to-face examinations: mid-clerkship examination and end-of-clerkship examination. Both examinations were composed of multiple-choice questions (MCQs) and objective structured practical examination (OSPE). The OSPE aimed to assess students’ radiologic interpretation skills. During OSPE, a series of cases (radiological images with pertinent clinical vignette) were presented in an automated manner (70 s for each case) using an overhead projector, and the student interpreted the image to answer the accompanying question.

Because of COVID-19 pandemic, our university has shifted to online distance learning and assessment. This created a challenge with summative assessment process, but it also created an opportunity to explore new methods of assessment. To create a successful and reliable online summative assessment, we as the examination moderator and department head had to increase the number and types of assessment tools that we utilize. This served two purposes. Firstly, it ensured that the intended learning objectives of the clerkship were broadly sampled and assessed with the proper tool. Secondly, it helped minimize negative impact should a student fail to complete one assessment tool, using a weighted score from the completed assessment forms can substitute for the missed one. Therefore, three online assessment tools were used: SOE, MCQ-type examinations, and homework assignments. The online SOE and homework assignments have replaced the OSPE for assessing students' radiologic interpretation skills. The online OSPE could not be administered due to technical difficulties. We conducted two simulated OSPEs online, one using Blackboard (Blackboard Inc., Washington, D.C., USA) and the other using Microsoft Forms (Microsoft Corporation, Washington, D.C., USA). These two platforms were utilized because they were made available by our university, and students must use their individual university login credentials to access them. Unfortunately, several students reported that images were loading slowly or failed to load entirely.

### Participants

All fourth-year medical students in our institution and the collaborating medical college who were enrolled in mandatory radiology clerkships in 2019/2020 academic year during the COVID-19 pandemic were included in this study. A total of 79 students took part in this study with 67 students from the first institution (Group A), and 12 students from the second institution (Group B).

### Faculty and students’ preparation for online SOE

Since shifting to online learning and assessment was a new experience, the faculty and students were prepared for the online SOE. We conducted training sessions to familiarize our faculty with Blackboard, and with virtual classrooms on Blackboard Collaborate and Zoom (Zoom Video Communications Inc., San Jose, California, USA). The students were trained on how to navigate through these online platforms and how to access and conduct online SOE. Mock examination was also conducted to familiarize the students and teachers with SOE. Explanatory multimedia-rich announcements were also sent to the students. The topics intended to be tested with SOE were outlined to the students.

### Development and execution of SOE

A SOE was constructed by three examiners with 1 to 7 years of experience in undergraduate radiology teaching. The examination was designed to test students’ competencies in interpretation skills and imaging appropriateness in common medical and surgical emergencies. It consisted of three clinically oriented cases. Each case had three predetermined questions and a detailed scoring guide. The scoring guide ensured consistent and fair grading for all students. Due to time constraints, only one online SOE was conducted. Given the importance of emergency imaging for future physicians, we wanted to carefully test students on it in the online SOE. Multiple other online assessment tools were used to test emergency imaging as well as the remainder of the clerkship learning objectives.

**Fig. 1:**
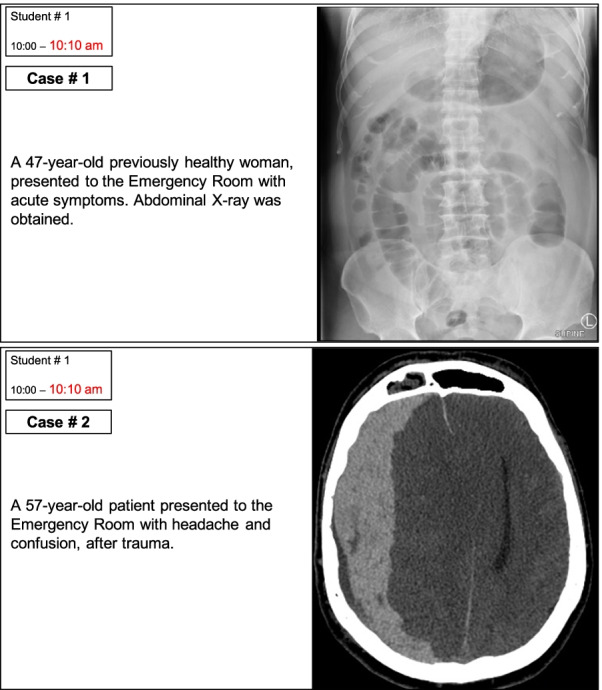
Sample questions (two of three cases) presented to a student. The left upper corner shows the order of the scheduled student (first students from each batch here) and start time and end time to easily keep track of students. The end time is highlighted in red to alert the examiner

**Fig. 2:**
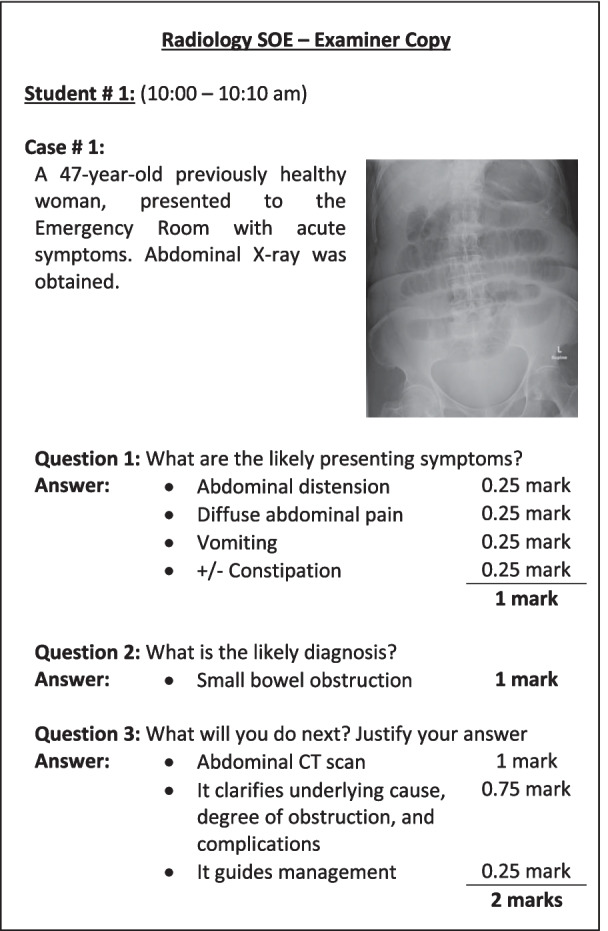
Sample of scoring guide given to the examiners. The order of scheduled student (first students from each batch here) and start time and end time are shown in the scoring guide to easily keep track of students

Five different versions of the SOE were created each with different set of questions. The SOE versions were then discussed with the other examiners in the radiology department to ensure clarity of the SOE questions and scoring guide, and uniformity in the degree of difficulty of questions among the different SOE versions. The fifth SOE version was used as a backup. Figure 1 shows two sample cases from one SOE version. Figure 2 shows the scoring guide for the first case. We used Microsoft PowerPoint (Microsoft Corporation, Washington, D.C., USA) to present the examination content to the students.

**Fig. 3:**
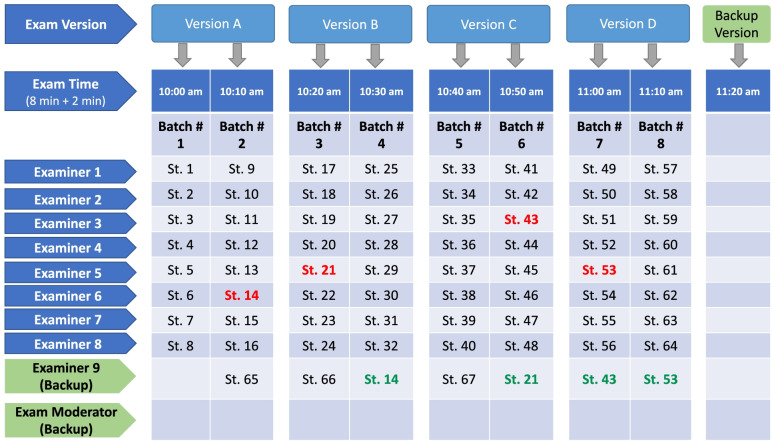
The SOE execution plan outlining the distribution (time, examination version and examiner room) for 67 students (St.1 to St. 67). Students highlighted in red (students # 14, 21, 43, and 53) had connectivity problems initially at their scheduled examination time, but they have been successfully re-allocated and examined in the backup examination room (highlighted in green). There was no need to utilize the second backup examination room (examination moderator room) and the backup examination version

The SOE was conducted via Blackboard Collaborate. Ten virtual oral examination rooms were created on Blackboard Collaborate. Eight rooms were assigned to primary examiners, while two rooms were considered backup rooms (Fig. 3). The two backup rooms were managed by two examiners (the examination moderator and another examiner). The backup examination rooms were used whenever any of the primary examination rooms encountered problems, such as technical difficulties. Students and examiners were instructed to contact the examination moderator in the event of a problem, and the moderator would facilitate the student's access to a backup examination room at a different time. To ensure easy and timely communication with students and examiners, the examination moderator provided his contact information (cell phone, WhatsApp, and email) to all students and examiners the day before the examination date.

Group A students were randomly placed into nine examination groups using the Random Enrollment feature on Blackboard. Students received the timetable the day before the examination date, illustrating which examination room to access, and at what time they must access it. Students were not allowed to access the examination rooms before their scheduled time. Students accessed the examination rooms with their unique username and password. When a student entered the virtual examination room, the examiner shared their screen and displayed the slides to the student (Fig. 1). The examiner and student interacted verbally, with the examiner asking specific questions that were predetermined in the scoring guide (Fig. 2). The student responded to the questions based on their interpretation of the provided image. The examiner recorded the student’s score using the scoring guide (Fig. 2). Each student was given 10 min in the room. Examiners were instructed to complete all questions within 8 min, with a 2-min grace period to compensate for any delay a student may have while accessing the examination room. The examination moderator sent reminders to the examiners 2 min before the end of the examination time. The allocation of 10 min per examiner was based on two considerations. First, because the online SOE covered only a small portion of the clerkship learning objectives, we believed that three cases (each with three questions) would be sufficient to assess these learning objectives, and that 10 min would be sufficient to complete these questions. Second, based on our prior experience with online lectures, we have observed that the longer the lecture, the greater the likelihood that students will encounter connection issues on our platform (data not published).

The first batch of students (8 students) accessed their respective examination rooms at 10:00 am. They were examined using Examination Version A. The second batch of students accessed their respective examination rooms at 10:10 am. They were examined using Examination Version A as well because interaction between the first and the second batches was unlikely. Likewise, batches 3 and 4 were examined using Examination Version B. The same procedure was used for batches 5 and 6 (Examination Version C) and batches 7 and 8 (Examination Version D). In total, 64 (out of 67) students were tested in the eight primary examination rooms, while the remaining 3 students were assigned into one of the backup rooms. Figure 3 shows the SOE execution plan.

**Fig. 4:**
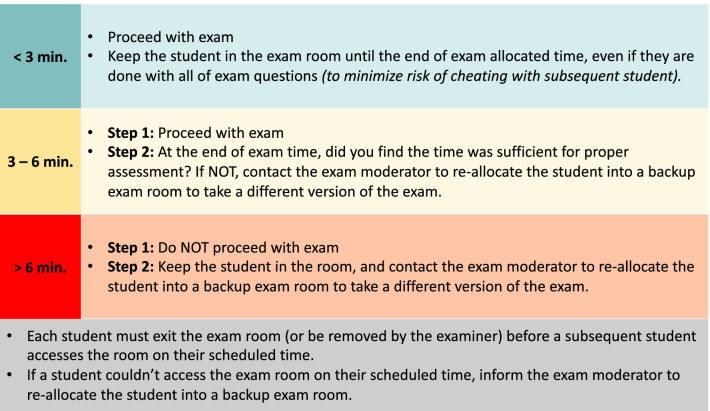
Instructions given to examiners clarifying three potential categories of students based on what time they would access the examination room and what actions should be taken. Appropriate action for students who could not access the examination rooms on their scheduled time is also clarified

Nine examiners participated in the SOE. Five examiners (senior examiners) had more than 5 years of experience in teaching and assessment of undergraduate medical students, while four examiners (junior examiners) had less than 2 years of experience. The examiners were given clear instructions on how to deal with dynamics of the examination and when to contact the examination moderator as shown in Fig. 4. The same process was followed for Group B students which included 12 students, using six primary examination rooms and a one backup examination room.

The final version of the examination and scoring guide was given and explained to the examiners the day before the examination date. The examiners were also instructed to contact the examination moderator immediately if they encountered any problems to be timely solved.

### Evaluation of the experience and outcome measurements

The number of students and examiners who encountered problems during the online SOE and the causes of the problems were recorded by the examination moderator. SOE percent scores were correlated with students’ prior academic performance (grade point average; GPA). SOE percent scores means were also compared between higher- and lower-GPA groups. Students’ GPA means were compared among different examination versions, and among examiners with different experience levels (junior and senior examiners) to ensure proper randomization and fair distribution of students. The SOE percent scores means were compared between the two institutions and between male and female students. To assess whether the distribution of questions difficulty among different SOE versions was fair, the SOE percent scores means were compared between various versions. Furthermore, the SOE percent scores means were compared between subsequent batches to assess if cheating was minimized. To evaluate the usefulness of the scoring guide in minimizing variations between different examiners, SOE percent scores means were compared among all examiners and between senior and junior examiners.

Students’ experience was assessed using an anonymous online survey conducted shortly after completion of the radiology clerkship and before awarding students’ grades. Two teachers, each with a 7-year experience in radiology clerkship teaching and assessment, created the preliminary survey. The preliminary survey was then pre-tested on a small group of the target students. Shortly after that, they were interviewed to further refine the survey [11]. As a result, several statements were changed and ambiguous statements were rephrased to ensure clarity. The survey was composed of 10 questions, including questions on individual students’ characteristics, students’ readiness for SOE (prior experience with online SOE, adequacy of students’ training for online SOE provided by radiology department, devices used, and use of webcam), and finally questions exploring the advantages and concerns regarding the use of online SOE. SurveyMonkey (SVMK Inc., California, USA) was used to build the survey.

### Statistical analysis

SOE scores, GPA, and survey data were registered on Microsoft Excel spreadsheet (Microsoft Corporation, Redmond, Washington). The descriptive and inferential statistics were calculated using the Excel data analysis tool. Pearson correlation coefficient (*r*) was used for correlation of SOE percent scores with GPA [12]. Differences in GPA means and SOE percent scores means between different groups were evaluated using unpaired student’s t test or ANOVA with post hoc Holm–Bonferroni procedure (to adjust *p* values and control for type I error). A *p* value of ≤ 0.05 was considered statistically significant.

## Results

### Participants

A total of 79 fourth-year medical students were enrolled in the radiology clerkships, and all were exposed to SOE. Group A, from one institution, included 67 students: 47 males (70.1%) and 20 females (29.9%). Group B, from another institution, included 12 male students. Nine examiners participated in the SOE. None of the examiners had prior experience with online SOE.

### Outcomes of online SOE

#### Success rate of online SOE execution

All students (*n* = 79) had successfully completed the SOE. Four students (5.1%; all from group A) experienced connectivity problems, which resulted in significant delayed entry into their examination rooms (> 6 min), but they were successfully re-allocated into the backup examination room within 10–30 min (mean = 17.5 min) of their original scheduled time (Fig. 3). Three students (3.8%; two from group A and one from group B) had moderate late entry (3 – 6 min), but it was deemed sufficient time for fair and adequate assessment by their examiners, and they did not require re-allocation into the backup examination room. The whole examination was completed within the scheduled time for both groups. None of the examiners had difficulty accessing their rooms, or difficulties during interaction with their students (i.e., sharing examination slides, and hearing and talking with the students).

#### Students’ GPA and SOE scores

**Fig. 5:**
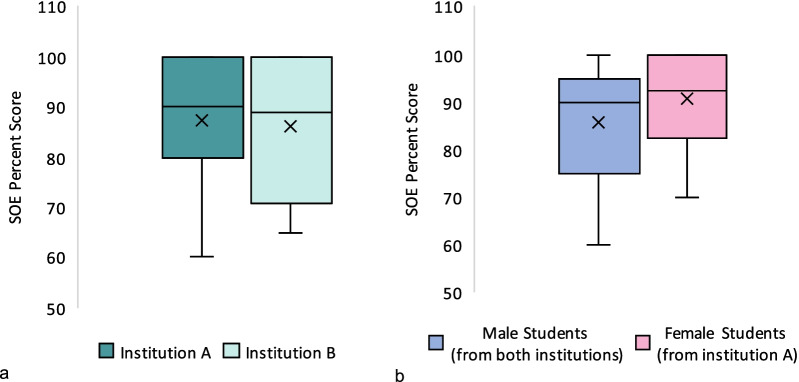
Comparison of boxplots of SOE percent scores between the two institutions (**a**) and between male and female students (**b**). There was no statistically significant difference between these groups

**Fig. 6:**
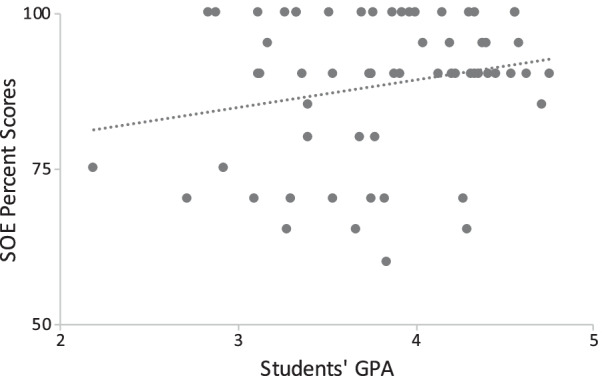
Scatterplot shows poor correlation between students’ GPA and their SOE percent scores

**Fig. 7:**
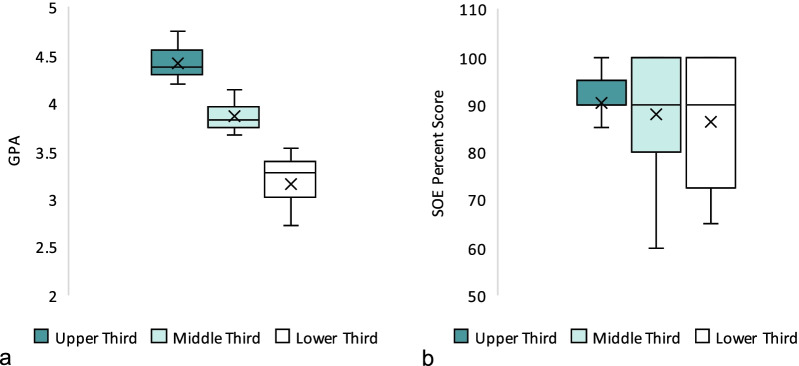
Comparison of boxplots of GPAs (**a**) and SOE percent scores (**b**) between three levels of students; upper third being students with highest GPAs, to lower third being students with lowest GPAs. The GPA means were significantly different between the three groups, while the SOE percent scores means were not

SOE percent scores ranged from 25 to 100, with a mean of 87.1 ± 13.6. SOE percent scores means were not significantly different between groups A and B (87.2 and 86.0, respectively; *p* = 0.781), nor between male and female students (85.8 and 90.8, respectively; *p* = 0.117) as shown in Fig. 5. GPAs for group A ranged from 2.10 to 4.76 out of 5.00; with a mean of 3.82 ± 0.57. Group B GPAs were not available to us. SOE scores showed poor correlation with the students’ GPA, with a Pearson correlation coefficient (*r*) = 0.22, and *p* = 0.09 (Fig. 6). The upper and lower thirds of students (i.e., those with highest and lowest GPAs, respectively) have GPA means of 4.42 ± 0.16 and 3.17 ± 0.32 out of 5.00, which showed statistically significant difference (*p* < 0.001). However, SOE percent scores were not statistically significant different between these two groups; 90.5 ± 8.8 versus 86.4 ± 12.8 (*p* = 0.238) as Fig. 7 shows.

**Fig. 8:**
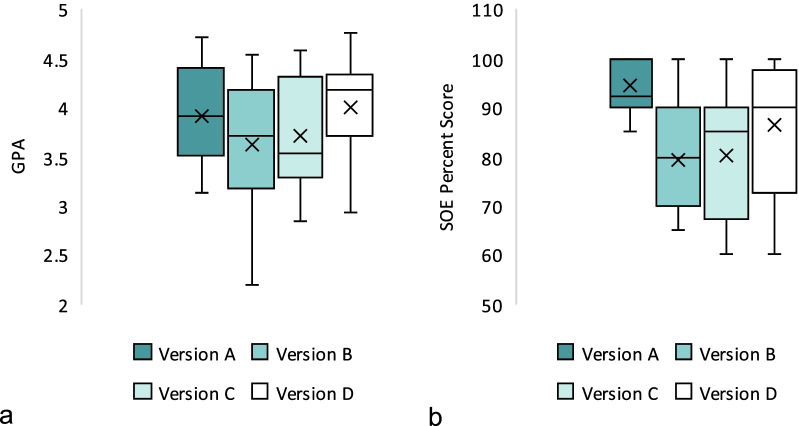
Comparison of boxplots of GPAs (**a**) and SOE percent scores (**b**) between four SOE versions. The GPA means were not significantly different between the four groups. Only SOE percent scores mean for version A was significantly higher than the other versions (*p* = .027); SOE percent scores means were not significantly different among the other three versions

Students who were exposed to examination versions A, B, C, or D have GPA means of 3.92, 3.63, 3.71, and 3.99 out of 5.00, respectively. There was no statistically significant difference among these groups (*p* = 0.218). Students’ percent scores, however, showed significant difference (*p* = 0.005) between the four versions. Post hoc analyses revealed that students who were exposed to version A (94.4 ± 5.4) have significantly higher scores than those exposed to version B (79.4 ± 17.8; *p* = 0.010) and version C (80.3 ± 12.2; *p* = 0.008) as shown in Fig. 8. Further analysis of the percent scores between two subsequent batches who had the same examination version (done for each examination version separately) showed no statistically significant difference between them.

**Fig. 9:**
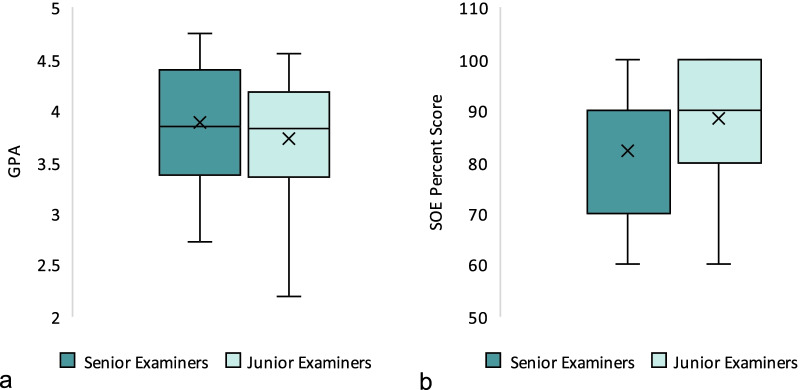
Comparison of boxplots of GPAs (**a**) and SOE percent scores (**b**) between senior and junior examiners. The GPA means and the SOE percent scores means were not significantly different between the two groups

The GPAs of students who were examined by junior examiners (3.73 ± 0.55) were not significantly different from those who were examined by senior examiners (3.89 ± 0.58; *p* = 0.269) as Fig. 9 illustrates. SOE percent scores were also not significantly different between the two groups (88.3 ± 11.5 and 82.3 ± 15.3; *p* = 0.078). During ANOVA analysis of students’ scores for different examiners (nine examiners, regardless of level of experience), a tendency for significance was found. However, on further post hoc analysis by multiple t tests with Holm–Bonferroni corrections applied, no significant differences were noted between the nine groups.

### Students’ perceptions of online SOE

A good response to the survey was achieved, with 81.0% response rate (*n* = 64/79), including 53 students from group A and 11 students from group B. Male and female student participants were 73.4% (*n* = 47/64) and 26.6% (*n* = 17/64), respectively. The participants’ age ranged from 21 to 30 years; mean 23.38 ± 1.43.

None of the students had any prior experience with online SOE. However, the majority of the students (*n* = 61, 95.3%) indicated that the radiology department efforts (mock examination and explanatory multimedia-rich announcements) helped them to become familiar with how to access and conduct online SOE, what to do, and whom to contact in case of problems, which made their experience easier. The majority of the students (*n* = 60, 93.8%) used computer, while 6.3% used tablets, and none of them used smart phones. The majority of the students (*n* = 46; 71.9%) agreed that using a webcam is acceptable, and it is the only reliable method for confirmation of student’s identity during online examinations. However, 23.4% of the students (*n* = 15) disagreed with using a webcam to confirm the student’s identity during the online examination; some citing that using the webcam would adversely affect their Internet connectivity and increase the examination anxiety.

Direct interaction with the examiner was considered an advantage of SOE by 38 students (59.4%). Two-thirds of the students (*n* = 44; 68.8%) indicated that online SOE is able to distinguish between excellent and poor-performing students. The majority of students (*n* = 41; 64.1%) found it stressful, and a minority found it difficult (*n* = 15; 23.4%), while another minority found it easy (*n* = 12; 18.8%). Concerns with SOE included anxiety from Internet connection issues at time of examination (*n* = 35; 54.7%), tight examination time (*n* = 13; 20.3%), and lack of experience with online SOE (*n* = 11; 17.2%). A minority of the students stated that there should be more than one examiner (*n* = 13; 20.3%).

## Discussion

Online assessment for feedback and formative assessment purposes have been used for years. It provides several potential benefits including flexibility in terms of place and time, facilitating instant feedback, motivating further study and understanding through links to additional learning resources, recording students’ activities and monitoring their progress, facilitating interaction between the students and teachers, and saving teachers time and efforts particularly when dealing with large groups of students [13–19]. On the other hand, the use of online assessment for summative assessment purposes has been rarely used. This is because of perceived risks of test security, increased cheating risks, authentication issues, limited examiner control, privacy issues, and accessibility hurdles [20, 21]. In a study done in 2016 in an Australian university [20], students were offered the opportunity to do their final examination online with invigilation via webcam, and they had the chance to do a trial. Only 6.3% of the students found it good experience and preferred to do their final examination online. The majority of the students did not like the experience of online summative assessment mainly because of technical difficulties [20]. In contrast with this finding, the experience with online SOE was found successful in our study despite being a new experience for instructors and students in our department. This is likely because the potential challenges were raised and ways to overcome them were discussed in advance.

The challenges encountered with online SOE in this study included, ensuring a successful, uninterrupted, and smooth implementation, maintaining examination integrity and minimizing chances of cheating, and reducing students’ anxiety with this new experience. To address the first challenge, we used contingency plans to successfully deliver online assessment. These contingency plans included setting backup examination rooms with backup examiners, having backup examination questions, and having a backup virtual platform (Zoom) available in case of failure of the primary platform (Blackboard). We achieved success rates of 94.9% and 100% before and after using the first backup strategy. To the best of our knowledge, the concept of backup examination rooms in online SOE had never been reported in the English literature before the pandemic. However, we had personal experience with pre-pandemic onsite radiology board oral examinations in which standby examiners were used as backup for primary examiners who were unable to attend the examination. After learning from this experience and anticipating Internet connectivity problems for examiners and students, we implemented backup virtual examination rooms. Recent studies have also reported the use of backup examiners in online examinations during the pandemic [22–24]. Clear and timely instructions, mock examination, and familiarizing the students and examiners with the online SOE process and platforms made the execution smooth. The online SOE execution, despite being a new experience, was well perceived by our students due to having contingency plans in place.

To address the second challenge, i.e., maintaining examination integrity and minimizing chances of cheating, we used four different examination versions. The versions were constructed using authentic clinically oriented questions that require image interpretation, rather than simple recall questions. The latter added another layer of security and ensured integrity because it would be difficult for the student to search a book or the Internet for answers within the examination time frame. Using multiple examination versions is a common examination security technique that has been used to prevent cheating, particularly in MCQ examinations [25–27]. However, using different examination versions is time consuming for the examiners, and it can result in variations in difficulty levels from one version to another [26, 27]. To mitigate this issue, each student was evaluated by one examiner instead of two examiners. This process resulted in increasing the number of simultaneously tested students, while reducing the number of examination versions provided. A minority of the students expressed preference for having two examiners to minimize subjectivity and variations between examiners. This is possibly a valid concern. However, assigning two examiners in our module would result in having four examination rooms instead of eight, 16 batches instead of eight, and 8 examination versions instead of four. All of this would increase the load on examiners and possibly increase variations among examination versions. To achieve a balance between examination security (which is enhanced by using multiple versions) and examination fairness (resulting from difficulty variation between versions), we opted to use one examiner for each student to minimize the number of examination versions needed. Additionally, the examination versions were evaluated by all examiners in advance to ensure having similar difficulty levels. An examination scoring guide with specific items and scores was also used to minimize inter-examiner variations. This is supported by the findings of Besar et al. [28] who found a significant strong inter-rater agreement (0.83–0.88) when using well-structured examinations with clear and specific items and scores in the checklist. They concluded that one examiner is sufficient for such examinations [28]. Clinical examinations that use structured checklists were found to minimize examiners’ subjectivity and variability [29]. Standardized scripted questions, which we used, are a recognized means for improving oral examination reliability as well [3]. Moreover, Burchard et al. have also found that raters’ experience did not significantly affect examinees’ scores on structured oral examination [30]. Our students’ results are in line with these conclusions as our students’ scores showed no significant difference between various examiners, and between junior and senior examiners. We also did not find significant difference in students’ scores between the two institutions or between male and female students. All of these results emphasize the usefulness of the scoring guide in minimizing variations among different examiners. Except for one examination version, which may be slightly easier, all other versions showed no significant difference in students’ scores, indicating having similar difficulty. This result also supports the above-mentioned conclusion which indicates that having one examiner for each student can reduce the number of examination versions used. We also found no significant difference in students’ scores between each two subsequent batches who were exposed to the same examination version. This supports the conclusion that cheating on examinations among the two batches of students had not happened; otherwise, the second batch students would have had higher scores.

Although two-thirds of the students (68.8%) perceived online SOE to be able to distinguish between excellent and poor-performing students, the students’ actual scores on examination did not support this conclusion. Students’ scores showed poor correlation with their GPAs. Although this is surprising given the strict measures taken against cheating as discussed earlier in the study, this finding is possibly attributed to a phenomenon known as “content specificity” or “case specificity” problem [29, 31, 32]. This problem is seen when an examination samples limited content of the learning objectives. The online SOE in our cohort tested only a small portion of the clerkship learning objectives, i.e., interpretation skills and imaging appropriateness in common medical and surgical emergencies. We will probably get different results if we use more SOEs and sample more clerkship content. Newble [29] recommends using other assessment methods to achieve broad content sampling to address this problem. Due to time constraints, we only conducted one online SOE. To ensure a comprehensive sampling of the clerkship learning objectives, we used multiple other online assessment tools, including multiple MCQ-type examinations and online homework assignments (not covered in this article). As a result, the overall students’ scores showed a moderate correlation with their GPAs. Another contributing factor to this “content specificity” problem was having three cases in each examination version with three questions on each. Instead, we could have increased the number of cases and reduced number of questions on each case. Prior literature suggested that any clinical problem has one or more “key elements,” and there are other elements which follow from these key elements and carry less importance. Therefore, it was advised to limit the assessment to the key elements to save time and enable testing for more problems, and eventually improve examination reliability [31, 33, 34]. For future online SOE, we intend to increase the number of cases (instead of the three cases we had) by increasing the examination time or by limiting the assessment to the “key elements” in order to save time and enable testing for more problems, and ultimately improve examination reliability. The SOE validity (content validity) was addressed at the time of examination items construction as two examiners authoring the cases had more than 7 years of experience, and the items were matched with clerkship blueprint. Furthermore, the final examination versions were reviewed with the rest of radiology department members to ensure that examination items indeed test the expected competencies.

Virtual proctoring which facilitates supervising examinations in real time using webcam is on the rise particularly for high-stake examinations [35]. Despite the advantages of virtual proctoring which include maintaining online examination integrity and academic honesty, and eliminating the need for test centers and physical proctors, it poses some challenges including implementation and operational issues, and examinees’ privacy concerns [36]. Using webcam monitoring for authentication and proctoring students can improve examination integrity and eliminate cheating, but this is not always a feasible option, like in our cohort where Internet issues and non-availability to all students have precluded this option. A recent study from Netherlands has also shown substantial negative impact of technical hurdles that students experienced with the virtual proctoring software [35]. Biometric (physiological or behavioral) characteristics have been used for authentication, and they are more secure and reliable than personal identification number and password [37, 38]. However, we only used university identification username and password access for authentication due to cost and time constraints associated with other authentication methods. Despite such technical challenges, we applied strategies (e.g., using authentic clinical interpretation questions, and using multiple examination versions) in order to minimize the risk of cheating.

Research has shown that anxiety can have a negative impact on performance and cognition [39, 40]. Apart from normal anxiety associated with any examinations, extraneous causes of anxiety in our students stem from anxiety of failure to conduct the examination, which is mainly because of Internet connectivity issues, and anxiety from the new experience with online SOE. Therefore, to alleviate students’ anxiety, familiarizing students and examiners with the examination process in advance was a key factor. This included training on the platforms used to conduct the examination, familiarizing them with the examination format, and having direct and immediate contact with the examination moderator via phone. Furthermore, during the examination, students who encountered connectivity problems were immediately dealt with and re-allocated into other examination slots with a backup examiner to reduce their worries. Despite the lack of prior experience with online SOE, the vast majority of students (95.3%) found orientation and training provided by the radiology department was very helpful to prepare them for online SOE. Nearly half of the students (54.7%) were anxious that their Internet connection may fail them during the examination. However, the contingency plans we implemented ensured that such an issue was solved instantly. Similarly, Justaniah et al. [7] have reported that majority of the examinees were anxious during the online SOE. They reported having technical difficulties with 42.9% of the examinees. This contrasts with our results where technical difficulties were limited in our cohort (8.9%). This is likely because we had shorter examination time (10 min versus 60 min) and a larger sample size.

## Study limitations

Using one SOE only may render the results non-generalizable. This is likely the main reason for the poor correlation between students’ GPAs and scores and the limited ability to distinguish between excellent and poor-performing students. Nevertheless, we showed that online SOE is feasible, cheating can be reduced, and fairness can be preserved regardless of examiners’ level of experience or using different examination versions. Increasing the number of cases within each SOE, doing more than one SOE, or doing other test formats will achieve broad content sampling and will likely improve assessment reliability. The inability to use webcam to proctor the SOE and confirm students’ identity may cast doubts on examination integrity and academic honesty. However, we opted not to use it because it was not available to all students, and live streaming will likely compromise the network connectivity during examinations, which may have more negative impact on the students. Unfortunately, our examination was not recorded. Recording the examination is an important step that we should have taken to address potential disputes. We intend to use it and recommend it in future online SOE.

## Conclusions

The study showed that radiology online SOE is a feasible alternative when traditional proctored SOE is difficult to do, for any reason. Proper preparation is a key to success of online SOE. Constructing several versions of authentic examination material that test higher cognitive abilities (interpretation and judgment) will reduce cheating. Reliability can be enhanced by increasing the number of cases within the SOE, doing more than one SOE, or doing other test formats. Reviewing various examination versions by a group of examiners can enhance fairness in difficulty among different examination versions. Using a detailed scoring guide will improve fairness of examiners. Familiarizing students and examiners with the assessment process, setting up backup plans, and timely communication with students and examiners will ensure smooth implementation of the assessment process and alleviate students’ anxiety. Proctoring is advised if it will not adversely affect online SOE implementation (e.g., because of Internet connectivity limitations). Recording the examination is crucial for resolving potential disputes.

It should be noted that planning for online SOE is a tedious task for the examination moderator. Like in other online tests, questions in online SOE cannot be reused in future examinations which adds more burden on examiners. Therefore, our study concludes that face-to-face SOE should be the default option as it reduces the load on examiners by reducing the number of examination versions needed and limiting the chances of examination leakage. However, online SOE is a feasible alternative whenever face-to-face SOE could not be implemented.

## Data Availability

The datasets used and analyzed during the current study are available from the corresponding author on reasonable request.

## Abbreviations

COVID-19: Coronavirus disease 2019
GPA: Grade point average
MCQ: Multiple-choice question
OSPE: Objective structured practical examination
SOE: Structured oral examination
